# PREDICTORS OF DEPRESSION IN BLACK WOMEN WITH HYPERTENSION: A SECONDARY ANALYSIS OF HRS DATA

**DOI:** 10.1093/geroni/igae098.0457

**Published:** 2024-12-31

**Authors:** Lashawn Hutto, Michelle Odlum, Laurie Theeke

**Affiliations:** George Washington University, Washington, District of Columbia, United States; George Washington University, Washington, District of Columbia, United States; The George Washington University, Ashburn, Virginia, United States

## Abstract

Black women experience a prevalence of depression and hypertension, with symptoms of depression going unrecognized. They describe worthlessness and hopelessness but also emptiness, lack of control, mood swings, and disinterest, which are not part of routine screenings. The Black women’s schema prioritizes strength, caring for others, and avoiding discussing feelings. Along with barriers of distrust of providers, self-denial of symptoms, limited depression knowledge, stigma, and financial limitations, diagnosis and treatment may be delayed. This study describes predictors of depression in black women based on data from the Health and Retirement Study (HRS). We examined individual, social, and relationship differences between black women with and without hypertension and used stepwise logistic regression to identify predictors. The sample included 407 Black women aged 50 years and above. Nearly half (47.7%) reported hypertension. The majority (94.6%) were married and had varying education, with the majority of participants having high school or less (66.6%). Women with hypertension reported worse overall health (X2=43.999, df=4, p=0.000), more health changes (X2=16.724, df=2, p=0.000), higher effort (X2=10.379, df=3, p=0.016) and depression scores (X2=9.518, df=3, p=0.23), restless sleep (X2=18.907, df=3, p=0.000) and less physical activity (X2=10.981, df=4, p=0.27.). Predictors for depression were loneliness (OR=3.509, 95% CI=1.364, 9.027), sadness (OR=2.272, 95% CI=1.026,5.031), restless sleep (OR=2.093, 95% CI=1.237, 3.542), unhappiness (OR=0.430,95% CI=0.258,0.71) and psychological problems (OR=0.194, 95% CI=0.038, 0.979). Future implications include building relationships with organizations that support black women with depression and longitudinal analysis of HRS data to explain the role of loneliness on depression and hypertension in this population.

